# Adaptations in the Context of COVID-19: Application of an Implementation Science FRAMEwork

**DOI:** 10.1007/s43477-022-00048-1

**Published:** 2022-06-27

**Authors:** Erin C. Albrecht, Lindsay Sherman, Amanda Fixsen, Julie Steffen

**Affiliations:** Invest in Kids, 1775 Sherman Street, Suite 1445, Denver, CO 80203 USA

**Keywords:** COVID-19, Implementation science, Evidence-based programs, Fidelity, Adaptations

## Abstract

Invest in Kids (IIK) is a Denver, Colorado, USA-based intermediary organization that works to bridge the research-to-practice gap for programs that support Colorado’s youngest children and their families. IIK supports evidence-based programs (EBPs) including three universal, prevention programs from The Incredible Years® (IY)—Dinosaur School (a classroom curriculum), Teacher Classroom Management (teacher professional development training), and Parent Program (parent training). IIK employs staff (the IIK–IY Team) to deliver implementation supports such as training, coaching, and managing implementation teams with professionals at schools and community agencies who deliver IY programs throughout Colorado. The COVID-19 pandemic presented challenges and opportunities for practitioners using EBPs, and for the IIK–IY Team. The Framework for Reporting Adaptations and Modifications—Expanded, known as the FRAME (Stirman et al., 2019), was identified as a useful tool for documenting information about COVID-19-related adaptations to local sites’ delivery of the programs, IIK’s implementation supports, and IIK’s annual statewide evaluation of IY. This case study includes an in-depth description of the various adaptations made by the IIK–IY Team, highlighting specific examples that demonstrate how the FRAME can be used to support adaptations for numerous sites in a geographic region or state. This case study provides important lessons about what successful IY delivery and implementation supports looked like in the context of the ongoing COVID-19 pandemic, including a description of IY fidelity and program outcomes.

**Supplementary Information:**

The online version contains supplementary material available at 10.1007/s43477-022-00048-1.

## Introduction

A central challenge of delivering evidence-based programs (EBPs) in community settings is maintaining fidelity to the program model and achieving outcomes comparable to those found in carefully controlled research settings (Wandersman & Florin, 2003; Webster-Stratton, 2004). Fidelity is defined as the extent to which the practitioner adheres to the standards for EBP delivery, as established by the program developer (Mincic et al., 2009). Implementation science research has shown that without proper attention to and substantial support for program delivery and fidelity to the program model, it is unlikely that a program will yield the intended outcomes (Durlak & Dupre, 2008). Intermediary organizations bridge the gap between program developers and effective widespread dissemination of EBPs, providing the necessary implementation support to promote high-quality delivery of EBPs in community settings (Franks & Bory, 2015; Lang et al., 2017).

Invest in Kids (IIK) is a Denver, Colorado, USA-based intermediary organization that partners with communities statewide to adopt, implement, and successfully scale EBPs for Colorado’s youngest children and their families. IIK supports Nurse-Family Partnership®, Child First®, and three universal prevention programs from The Incredible Years® (IY)—Dinosaur School (a classroom curriculum), Teacher Classroom Management (teacher professional development training), and the Preschool BASIC Parent Program (Parent Program; parent training). The suite of IY programs focuses on strengthening parent–child, teacher–child, and home–school connections that provide a strong foundation for young children’s social and emotional development (Webster-Stratton, 2001). Over 40 years of randomized control trials and successful replication have documented the benefits of IY programs, which include greater social–emotional skills in young children, fewer conduct problems for children with high levels of non-compliant and aggressive behavior, the increased use of positive teacher classroom management strategies, and improved parenting practices (e.g., Webster-Stratton et al., 2001).

The Active Implementation Frameworks (Fixsen, Blase, et al., 2019; Fixsen, Van Dyke, et al., 2019) guide IIK’s comprehensive approach to working with and supporting sites throughout the implementation process. To that end, IIK employs IY-trained staff to provide high-quality implementation support for the IY programs, including training, coaching, managing implementation teams, and supporting professionals at schools and community agencies where the programs are delivered. The IIK–IY Team includes professionals with expertise in implementation science and evaluation (e.g., the IIK–IY Data and Evaluation Manager) who support ongoing technical assistance, annual IY evaluation, and routinely monitor program fidelity and outcomes in service to continuous quality improvement. IIK staff also works with state government, the philanthropic community, and public and private donors to secure funding and ensure long-term sustainability of the IY programs in Colorado.

Dinosaur School is a curriculum used by early childhood teachers in preschool and kindergarten classrooms that involves using child-sized puppets to help teach students social–emotional concepts and skills, such as how to identify feelings in themselves and others, how to calm down when angry, and how to solve social problems with peers. Other fidelity elements include showing vignettes, having students role play to practice new skills, leading large and small group activities, and sharing homework with families to promote students’ social–emotional development. The IIK–IY Team provides three days of Dinosaur School training to teachers spread out over a school year. The IIK–IY Team also provides six coaching visits for first-year Dinosaur School teachers and four coaching visits for second-year Dinosaur School teachers in order to support the many skills required to deliver the curriculum with fidelity. Teacher Classroom Management is a comprehensive training that the IIK–IY Team provides to preschool and kindergarten teachers and educational staff over five days throughout the school year. This program emphasizes building positive relationships with students and managing classroom behavior through the application of a hierarchy of discipline strategies. In addition to training, IIK–IY Team member provides five Teacher Classroom Management coaching visits throughout the year to further support teachers’ application of skills. In service to IIK’s mission, the IIK–IY Team prioritizes partnering with schools where there are relatively high rates of students who qualify for free or reduced lunch, and/or sites serving students who qualify for free or subsidized preschool.

Parent Program is a skill-building program for parents or guardians of preschool-aged children. The program is delivered in English or Spanish over 28 hours across 14 weeks to a group of approximately 7 to 15 participants and includes childcare and a meal for participating families at each weekly session. Facilitators receive training and coaching from the IIK–IY Team on how to deliver the program with fidelity, including showing vignettes, setting up role plays, and discussing the application of a hierarchy of discipline strategies with parents, which serves as the foundation for supporting children’s behavior at home. Parent Program aligns with Dinosaur School and Teacher Classroom Management in that parents and teachers learn to support the children’s social–emotional development by learning and applying an array of evidence-based strategies. Each of the three programs emphasize supportive, nurturing relationships between caregivers and children as the necessary foundation for children’s positive behavior, whether at home or in the classroom.

The purpose of this article is to provide a case study of the application of an implementation science informed tool, the Framework for Reporting Adaptations and Modifications—Expanded (Stirman et al., 2019), also known as the FRAME, that guided the IIK–IY Team’s planning and decision-making in response to the COVID-19 pandemic. This case study introduces IIK’s implementation support approach that is informed by the Active Implementation Frameworks (Fixsen, Blase, et al., 2019; Fixsen, Van Dyke, et al., 2019), offers context about COVID-19 and its implications for IIK’s work, and walks through the use of the FRAME and how the tool facilitated comprehensive documentation of the alterations that were made.

### Using the Active Implementation Frameworks in Practice

A critical feature of IIK as an intermediary to support IY programs is its use of implementation science to inform practice at every level—from exploration conversations with agencies prior to program delivery, to training and coaching teachers and Parent Program facilitators, to the ongoing collection and use of data by partner agencies and the IIK–IY Team. Partner agencies include school districts, Head Start sites, private non-profit preschool programs, local mental health agencies, Early Childhood Councils, and others that provide services to children and families who are typically in under-resourced settings. The Active Implementation Frameworks provide structure for IIK’s work with partner agencies. IIK draws heavily on the Implementation Drivers from the Active Implementation Frameworks and, in combination with each unique agency context and capacity, applies the Drivers in an integrated and compensatory manner. The Implementation Drivers have been widely demonstrated to correlate with successful implementation and socially significant outcomes (Fixsen, Blase, et al., 2019; Fixsen, Van Dyke, et al., 2019). See Online Resource 1 for examples of the Implementation Drivers used at IIK.

### IIK–IY and the Context of COVID-19

During the 2019–2020 program year (i.e., school year), IIK partnered with communities in 24 of Colorado’s 64 counties to support Dinosaur School, Teacher Classroom Management, and Parent Program for 7133 students (i.e., preschool, kindergarten, and first grade), 516 teachers and educational staff, as well as 94 Parent Program facilitators and 746 Parent Program participants. At the onset of the COVID-19 pandemic in March 2020, the shift to remote work for IIK staff members, the closure of Colorado’s schools for in-person learning and transition to virtual learning for teachers and students, as well as public health mandates restricting in-person gatherings, had widespread implications for the delivery of the IY programs and IIK’s implementation supports. The IIK–IY Team led with flexibility and responsiveness to the evolving public health crisis and changing needs of program implementers and participants, including teachers, parents, and students. At the same time, the IIK–IY Team prioritized supporting the transition of program delivery to virtual spaces through the end of the program year.

In the early weeks and months of the pandemic, it was unclear how long it would be before IIK and the schools and community agencies across Colorado delivering IY would return to business as usual. In August 2020, as the IIK–IY Team was busy preparing for the 2020–2021 program year, it became clear that a longer-term plan for supporting and delivering the IY programs in a virtual world was necessary. This shift involved a meeting with the program developer to tailor the core elements of the IY programs to fit a virtual context, making alterations to IIK’s implementation support, and adjustments to IIK’s annual statewide evaluation of IY. Given the number of alterations needed, and the scale and pace at which alterations had to be supported, the IIK–IY Team wanted to remain thoughtful and intentional about planning for, measuring, and monitoring such alterations. As stewards of IY, an underlying goal for the IIK–IY Team was to make appropriate, responsive alterations while also maintaining a high level of fidelity. With an eye toward documenting the alterations for the 2020–2021 program year while continuing to monitor IY fidelity and program outcomes, the FRAME (Stirman et al., 2019) was identified as a useful tool for codifying information about COVID-19-related alterations to local sites’ delivery of the program, IIK’s implementation supports, and IIK’s annual statewide evaluation of IY.

The FRAME outlines nine key questions to consider as part of the alterations process (Stirman et al., 2019). The first question involves recording what was altered, including program content, context, training and evaluation, and/or implementation activities. If the alterations are to program content or context, there is room to further delineate. Another question is the level at which the alteration is captured, including whether it affects one client, one site, or the entire system in which the program is delivered. It is also important to catalog at what point during the implementation process the alteration was made. Reasons that the program was altered, including the overall purpose and contextual variables that played a role in the decision-making process, are essential to account for (Stirman et al., 2019).

There are several other questions intended to capture key aspects of the alteration process that may facilitate the understanding of how the alteration was enacted and the extent to which it was successful (Stirman et al., 2019). This includes identifying who was involved in the decision-making process surrounding the alteration and whether the alteration was planned and proactive, or unplanned and reactive. It is important to note that Stirman et al. (2019) use the term “adaptation” to describe intentional, planned alterations. The term “modification” is used more globally to refer to any type of alteration that occurs, regardless of the level of proactivity or forethought for making such a change (Stirman et al., 2019). Lastly, whether the alteration is aligned with fidelity (if known) is crucial for understanding whether a similar level of program effectiveness and outcomes can be expected (Stirman et al., 2019). In the following section, we showcase specific examples of how the IIK–IY Team used the FRAME’s key questions as an anchor for a proactive, purposeful planning and decision-making process about alterations for the 2020–2021 IY program year. Throughout this case study, the alterations that the IIK–IY Team made to the program, IIK–IY’s implementation supports, and the annual evaluation will be referred to as adaptations, consistent with Stirman and colleagues’ (2019) definition.

## Case Study: IIK–IY and the Application of the FRAME

This case study documents the adaptations to Dinosaur School (see Table 1) and Parent Program (see Table 2) as a consequence of the ongoing COVID-19 pandemic and its effects on the contexts in which both programs were delivered. The IIK–IY Team did not support Teacher Classroom Management during the 2020–2021 program year and thus it will not be discussed here. Several aspects of the adaptations that the IIK–IY Team made were applicable to both Dinosaur School and Parent Program, regardless of whether the adaptation was to the program itself, IIK–IY implementation supports, or the annual evaluation. In terms of when the adaptations occurred, the 97 sites that delivered Dinosaur School as well as the 21 sites that delivered Parent Program during the 2020–2021 program year were in the implementation phase. As the IIK–IY Team engaged in a proactive adaptation planning and decision-making process prior to the start of the 2020–2021 program year, it was mostly unclear to what extent the adaptations, either individually or collectively, would impact fidelity and the corresponding program outcomes. Although the answers to that question are beyond the scope of the IIK–IY evaluation and the current case study, adaptations to the evaluation, which included the corresponding tracking of program and implementation support adaptations, enabled IIK’s measurement of IY fidelity and proximal outcomes in the context of the pandemic. A description of the program fidelity and outcomes from the 2020–2021 IIK–IY evaluation follows each summary of the Dinosaur School and Parent Program adaptations.

**Table 1:** 2020–2021 IIK–IY Dinosaur School adaptations overlay with FRAME questions (Stirman et al., 2019)

What was modified?	Additional information about what was modified?	For whom/what is the modification made?	Nature of the content or contextual modification?	Who participated in the decision to modify?	What was the goal?
Context/Program delivery	Some teachers and students participated in Dinosaur School via virtual or hybrid classroom settings	Dinosaur School teachers and students	Format, setting	IIK–IY Team, Program Developer	Improve feasibility
Evaluation/Dinosaur School fidelity checklist	Rating scale condensed, some items tailored to reflect virtual program delivery	IIK–IY Team uses the Dinosaur School Fidelity Checklist internally and with Dinosaur School teachers	N/A	IIK–IY Data and Evaluation Manager, IIK–IY Team	Improve feasibility
Evaluation/Student outcomes	Student outcome measures completed by teachers based on program delivery format	Dinosaur School teachers	N/A	IIK–IY Data and Evaluation Manager, IIK–IY Team	Account for program context
Content/Dinosaur School lessons	Tailored and added content to lessons to increase relatability for children and ease of use for teachers during pandemic	Dinosaur School teachers and students	Adding elements, tailoring	IIK–IY Team, Program Developer	Increase engagement, Improve fit with context
Context/Coaching for year 2 Dinosaur School teachers	Classroom observations and teacher coaching were done virtually, rather than in person	The IIK–IY Team, and the Dinosaur School teachers receiving coaching	Format, setting	IIK–IY Team	Improve feasibility, improve effectiveness of program

**Table 2:** 2020–2021 IIK–IY Parent Program adaptations overlay with FRAME questions (Stirman et al., 2019)

What was modified?	Additional information about what was modified?	For whom/what is the modification made?	Nature of the content or contextual modification?	Who participated in the decision to modify?	What was the goal?
Context/Program delivery	The program was widely available for participants via virtual settings	Parent Program facilitators and participants from virtual groups	Format, setting	IIK–IY Team, Program Developer	Improve feasibility
Evaluation/Parent Program fidelity checklist	Rating scale condensed, some items tailored to reflect virtual program delivery	IIK–IY Team uses the fidelity checklist internally and with Parent Program facilitators	N/A	IIK–IY Team	Improve feasibility
Evaluation/Participant outcomes	Questions related to the program delivery context added to the parent satisfaction survey	Parent Program participants	N/A	IIK–IY Data and Evaluation Manager, IIK–IY Team	Account for program context
Evaluation/Facilitator feedback	Facilitators surveyed about virtual versus in-person group experiences	Parent Program facilitators	N/A	IIK–IY Data and Evaluation Manager, IIK–IY Team	Account for program context
Content/Weekly parent group sessions	Shortened duration, reduced amount of program content and activities	Parent Program facilitators and participants from virtual groups	Shortening/condensing (pacing/timing), Removing/skipping elements, Loosening structure	IIK–IY Team, Program Developer	Improve feasibility, maintain reach and engagement
Context/Coaching	Parent group observations and coaching of facilitators done virtually	Parent Program facilitators	Format, setting	IIK–IY Team	Improve feasibility, improve effectiveness of program

### Dinosaur School Program Adaptations

Adaptations to Dinosaur School for the 2020–2021 program year involved contextual changes, with Dinosaur School teachers completing lessons with students in person, virtually, or using a hybrid format depending on the instructional format for a given school district, classroom, or student. The IIK–IY Team was committed to supporting teachers to deliver Dinosaur School, regardless of their instructional format. The intended, in-person Dinosaur School instructional setting allows students to physically interact with the teacher, Dinosaur School puppets, and other children during lessons, whereas students participating virtually were limited in their access to materials and interactions with peers, puppets, and their teacher. The goals underlying the Dinosaur School contextual adaptations were to improve feasibility of program delivery for teachers, as well as to increase the reach of the program to students and teachers whose only option was to engage in virtual or hybrid instruction.

A second planned adaptation was to add program elements by creating supplemental Dinosaur School lessons and visual resources that were responsive to how students’ and teachers’ learning contexts were altered amid the ongoing pandemic, such as how to recognize feelings when wearing masks and understanding physical distancing. These materials were developed by the IY–IIK Team and the IY program developer. The IIK–IY Team also created tip sheets with guidance and best practices for teachers about delivering core fidelity elements virtually, in person, or using a hybrid approach. The goals of adding supplemental Dinosaur School lessons and resources were to increase student and teacher engagement with the program and to improve the fit of the program content with a virtual program delivery context.

### Dinosaur School Implementation Support Adaptations

During the summer of 2020, it became clear that due to updated school policies, in-person training and coaching for Dinosaur School teachers would not be possible. The IIK–IY Team decided to offer virtual coaching, but only for second-year Dinosaur School teachers, who had already completed in-person training (i.e., before the pandemic shut down schools in March 2020). The goal of modifying the coaching context was to improve the feasibility of coaching despite virtual requirements and to improve the effectiveness of the program in the virtual context. The contextual adaptation to the coaching format and setting was to shift exclusively to virtual observations and coaching, which meant that there was no real-time, in-person modeling of concepts with the teacher.

### Dinosaur School Evaluation Adaptations

For the IIK–IY process evaluation, the shift to virtual coaching and the use of in-person, hybrid, and/or virtual delivery formats for teachers during the 2020–2021 program year led to consideration of whether adaptations were needed in order to resume assessing teachers’ Dinosaur School fidelity. The intent of the IIK–IY Team was to assess adherence to the core program elements and monitor teacher fidelity throughout the program year across all program delivery formats.

Prior to the onset of the pandemic, the IIK–IY Dinosaur School fidelity checklist was used, consisting of 18 fidelity items that were rated on a 5-point Likert scale, with higher scores indicating a greater degree of fidelity to each item on the checklist. This unpublished checklist was previously developed by the IIK–IY Team to assess and support ongoing monitoring of teachers’ fidelity. There was a rating scale and a series of guide statements for each item, and the score for each fidelity item was based on the number of guide statements the IIK–IY Team member observed as completed by the teacher during the coaching visit. Example items from the fidelity checklist include “Teacher uses developmentally appropriate content in Dinosaur School large group,” and “Teacher integrates Dinosaur School visuals and/or social–emotional visuals and materials into their large group Dinosaur School lessons.” The IIK–IY Team completed this checklist three times throughout the school year (at the beginning, middle, and end of the program year, approximately).

For the 2020–2021 program year, the IIK–IY Team made the decision to revise the existing Dinosaur School fidelity checklist in order to improve the fit of the checklist for assessing teacher fidelity across different program delivery formats. The proactive process to update the Dinosaur School fidelity checklist involved the IIK–IY Team examining each fidelity item and the corresponding guide statements, and identifying which fidelity items and guide statements could be assessed regardless of program delivery format. Most of the fidelity items and guide statements already met this criterion. For the few fidelity items and guide statements that did not meet this criterion, the wording was tailored so that they could still be evaluated across each program delivery format. The IIK–IY Team also identified a small number of guide statements that were only applicable to in-person program delivery, and this information was indicated in the updated fidelity checklist. In addition, a couple of the Dinosaur School fidelity items were reformatted as open-ended questions, in order to gather qualitative information about what teacher delivery of that item looked like across the different program delivery formats.

With the goal of making it easier for the IIK–IY Team to rate teacher fidelity, it was decided that the fidelity checklist rating scale would also be revised. Rather than using a 5-point Likert rating scale based on the number of guide statements observed, there were instead two dichotomous response options for each fidelity item. The first question was whether the fidelity item was observed, with the response options, “yes” or “no.” The second question was to what extent the fidelity item was delivered, with the response options “full” or “partial.” “Full” indicated that all of the guide statements were completed for that fidelity item, and “partial” indicated anything less than all guide statements had been completed. The modified Dinosaur School fidelity checklist consisted of 20 total items that were fairly similar to the previous checklist, with the revised rating scale being the most substantial adaptation. See Online Resource 2 for a list of the 2020–2021 IIK–IY Dinosaur School fidelity items.

Another aspect of the IIK–IY process evaluation involves the IIK–IY Team’s coaching visits. These coaching visits and key details are recorded by the IIK–IY Team and tracked in an online platform. Two questions were added to the 2020–2021 visit tracking forms to capture essential details about the program delivery format and virtual coaching at each visit with second-year Dinosaur School teachers. The first question was about the specific virtual coaching modality used to support the teacher (e.g., Zoom, Google Meets, Microsoft Teams, etc.) and the second question was about the teacher’s program delivery format (in-person, virtual, hybrid). The goal of adding these questions was to monitor Dinosaur School coaching visits, accounting for program delivery format.

The variability in program delivery format also necessitated adaptations to the IIK–IY Dinosaur School student outcomes evaluation. As part of this annual pre-post evaluation, teachers complete demographic information about each student, and two different measures of student outcomes at the beginning and end of the program year. The Dinosaur School student pre–post-outcome measures include 25 items that make up the Social Competence Scale-Teacher (SCS-T) Report (Conduct Problems Prevention Research Group, 1995) and nine additional items that were developed by the IIK–IY Team to assess the development of students’ specific, curriculum-related skills. These nine items are collectively referred to as Dinosaur School Social–Emotional Skills measure. Example items from the SCS-T include “This child can wait in line patiently when necessary,” and “This child shares materials with others,” and examples from the Dinosaur School Social–Emotional Skills items include “This child uses Dinosaur School language frequently and consistently,” and “This child is visibly engaged and excited to participate in Dinosaur School lessons.” The SCS-T was completed by teachers of students who were receiving in-person or hybrid instruction, and the Dinosaur School Social–Emotional Skills measure was completed by all teachers, regardless of instructional format, on a scale of one to five, with higher scores indicating a greater degree of social competence and greater use of Dinosaur School social–emotional skills in the classroom. Based on the anticipated variability in program delivery formats throughout the program year, even within the same classroom, the evaluation included asking teachers about the Dinosaur School format for each student at both the beginning and end of the school year. The goal of gathering this information for each student was to be able to examine student-level outcomes by program delivery format.

Prior to the fall 2020 pre-test with Dinosaur School teachers, the IY Data and Evaluation Manager reflected on ways to help ensure the validity of teachers’ responses to the IIK–IY outcome measures. This process included considering whether teachers would be able to observe students’ behavior in person and/or virtually for both pre-test measures. The IIK–IY Team ultimately decided that the teachers delivering Dinosaur School in person or using a hybrid model would complete both measures for each of their students. All teachers, regardless of program delivery format (i.e., including virtual program delivery), would be asked to fill out the second, shorter measure for each of their students.

Ahead of the fall 2020 pre-test data collection, the IY Data and Evaluation Manager added questions to the pre-test about teachers’ plans for Dinosaur School program delivery during the 2020–2021 school year. These questions were designed to gather information about the timing, frequency, and format of Dinosaur School delivery at the start of the school year. Questions were also added that asked about how often teachers planned to deliver the core Dinosaur School fidelity elements. In the spring of 2021, the IY Data and Evaluation Manager added questions to the teacher post-test that requested qualitative feedback about successes and challenges delivering the Dinosaur School program during the 2020–2021 school year. Additional questions designed to capture the context of program delivery were added to the post-test, including the teaching format that was used for the majority of the school year (i.e., 6 months or more), whether teachers and students had to transition between in-person and virtual learning at any point throughout the school year, and the Dinosaur School delivery format.

### Dinosaur School Outcomes and Fidelity

It is important to note that IIK routinely collects IY outcomes and fidelity data as part of a continuous quality improvement process. The information presented in this case study is based on de-identified data and analyses that were previously performed and documented as part of the 2020–2021 IIK–IY evaluation in Colorado. Thus, there was no review by an Institutional Review Board. The IIK–IY Team partnered with Colorado communities across 13 counties to support the delivery of Dinosaur School for 4027 students and 315 teachers during the 2020–2021 program year. See Online Resource 3 for demographic information about the 2020–2021 Dinosaur School students and teachers.

Table 3 demonstrates the Dinosaur School student outcomes by program delivery format. As part of the 2020–2021 IIK–IY Dinosaur School evaluation in Colorado, a paired samples *t*-test analysis revealed that, on average, there was a statistically significant difference between the pre- and the post-test for the Dinosaur School SCS-T overall score and the Dinosaur School Social–Emotional Skills score, for all program delivery formats. On average, students who received virtual, hybrid, or in-person Dinosaur School lessons demonstrated significantly higher levels of social competence and Dinosaur School curriculum-related skills at the end of the school year, compared to the beginning of the school year. The size of the pre–post-difference for the SCS-T overall score was quite similar, on average (within a tenth of a point), for students who had received hybrid or in-person Dinosaur School for the majority of the school year. The size of the pre–post-difference for Dinosaur School Social–Emotional Skills yielded an overall score that was within a tenth of a point, on average, regardless of whether the program format was virtual, hybrid, or in-person. The adaptations to the Dinosaur School outcomes evaluation enabled IIK’s measurement of students’ Dinosaur School outcomes in context.

**Table 3:** 2020–2021 IIK–IY Dinosaur School student outcomes by program delivery format

Outcome variable^a^	Program delivery format^b^	*M* (SD)	*t*^c^	df	*p* value
Social Competence overall score (SCS-T)	Virtual	N/A	N/A	N/A	N/A
	Hybrid	− 0.74 (0.65)	− 15.38	185	< .001
	In person	− 0.78 (0.71)	− 54.61	2493	< .001
Dinosaur School Social–Emotional Skills Items overall score	Virtual	− 0.83 (0.96)	− 21.72	637	< .001
	Hybrid	− 0.89 (0.68)	− 23.07	310	< .001
	In person	− 0.85 (0.74)	− 58.71	2653	< .001

*SCS-T* Social Competence Scale-Teacher Report

^a^Items for both variables are rated on a scale of 1 to 5

^b^Program delivery format indicates the format by which the student received the program for the majority of the school year (i.e., 6 months or more)

^c^Paired samples (pre–post) *t*-test results by program delivery format for the 2020–2021 IIK–IY Dinosaur School evaluation in Colorado

The assessment of Dinosaur School teachers’ successes and challenges during the 2020–2021 program year provides additional context to the quantitative student outcomes, as well as the program adaptations. A content analysis of teacher’s open-ended feedback revealed that overwhelmingly, teachers perceived a high level of student engagement during Dinosaur School lessons, even for students who participated virtually. Regardless of program format, teachers described how much growth they saw in students’ Dinosaur School-related skills, including the ability to regulate emotions, use Dinosaur School language, and solve problems. Teachers also shared their perception that in some cases, the program helped to alleviate students’ challenging behaviors. Some noted that the Dinosaur School program content was especially well suited for supporting students’ social–emotional development in the context of the pandemic.

Though some teachers mentioned that they experienced success delivering the program virtually, a considerable number of them indicated that delivering the program virtually, and/or having to transition between in-person and virtual program delivery was a challenge. In particular, it was difficult for some teachers to figure out how to use technology and deliver some of the fidelity elements virtually, such as small group lessons and role plays. Regardless of program delivery format, teachers noted that the overall context of the pandemic was taxing, as there were related issues with staffing, student attendance, and challenging student behaviors. Many teachers also reported less time to spend on Dinosaur School, which affected the frequency and duration of the lessons. Taken together, the transition to virtual Dinosaur School and related issues delivering the program in this context were the biggest barriers for teachers, but they also reported a great deal of success in terms of students’ continued engagement with the program and their growth in curriculum-related skills. It seems that teachers experienced more difficulty maintaining fidelity delivering the program virtually, but they still saw positive outcomes for students, which is consistent with the quantitative student pre–post-outcomes.

Adaptations to the IIK–IY Dinosaur School fidelity checklist allowed the IIK–IY Team to monitor Dinosaur School teachers’ fidelity at three different timepoints throughout the 2020–2021 school year. The number of teachers that were observed delivering the program in person increased at each checklist, which reflects the increase in the number of schools across the year that transitioned back to in-person learning. Overall, there were a small number of teachers observed doing virtual or hybrid Dinosaur School compared to the number of teachers that were observed delivering the program in person at each timepoint. On average, the IIK–IY Team observed roughly 83% of all fidelity items for in-person Dinosaur School teachers at each checklist. For teachers delivering Dinosaur School virtually, the IIK–IY Team observed, on average, 76% of fidelity items at checklist one, 73% of fidelity items at checklist two, and 72% of fidelity items at checklist three. On average, the IIK–IY Team observed about 80% of the fidelity items at checklist two and 94% of the items at checklist three for teachers that were using a hybrid model to deliver Dinosaur School. See Table 4 for the mean number of Dinosaur School fidelity items observed (i.e., “yes” for that fidelity item) by program delivery format.

**Table 4:** 2020–2021 Mean number of IIK–IY Dinosaur School fidelity items observed by program delivery format

Program delivery format^a^	Checklist 1^b^		Checklist 2		Checklist 3	
	*n*^c^	*M*	*n*	*M*	*n*	*M*
Virtual	11	13.64	6	13.17	2	13.00
Hybrid	0	–	4	14.50	2	17.00
In person	26	15.00	30	15.27	32	15.44

^a^Program delivery format indicates the format the teacher was using to deliver the program when each fidelity checklist was completed

^b^There were a total of 18 items on the IIK–IY Dinosaur School Fidelity Checklist included in the mean fidelity ratings

^c^Refers to the number of teachers for whom the IIK–IY Team had completed fidelity checklists

### Parent Program Adaptations

Parent Program adaptations for the 2020–2021 program year involved contextual changes, with Parent Program facilitators meeting with groups of participants in person or virtually, depending on the policies of the host agency and local COVID-19 guidance. At the start of the 2020–2021 program year, almost all of the Colorado schools and agencies that were hosting Parent Program planned to offer it virtually, and in consultation with the IY program developer, the IIK–IY Team remained committed to supporting Parent Program. Accordingly, the setting in which families were virtually engaging with the program was at home, instead of attending the in-person group at a common location, such as a school or trusted community agency.

There were several adaptations to the program content; all were promoted by the program developer during summer 2020. First, virtual weekly sessions were condensed and shortened to 90 minutes, in contrast with two hours for an in-person session. With less time for the Parent Program facilitators to cover each week’s topics and objectives, the amount of content and activities covered during each session was reduced. For example, two of the core fidelity elements for Parent Program involve video vignettes that showcase examples of different parenting scenarios, and role plays where parents apply program strategies. Due to the shorter duration of each virtual session, parents were exposed to fewer vignettes and role plays. There was also a reduced group size requirement, with only six to eight Parent Program participants for a virtual group, in contrast with 7 to 15 participants for in-person groups. Given the pervasive and traumatic nature of the ongoing pandemic, facilitators were also encouraged to spend additional time checking in on families, emphasizing self-care and stress management. Thus, the structure of the group sessions was loosened to accommodate time spent checking in with families.

During the IIK–IY Team’s discussions with the program developer, it was determined that a couple of the core fidelity elements, including the provision of meals and childcare for families, would be markedly different for virtual groups. Prior to the onset of the pandemic, in-person groups provided a free, family-style meal for the Parent Program facilitators, Parent Program participants, and their children, and free childcare was available on site for families to use while they participated in the group. Although the program developer was aware of the need to be flexible with these fidelity elements, it was also acknowledged that the removal of these elements was inconsistent with the program model. Nevertheless, the goals of the IIK–IY Parent Program content and contextual adaptations were to improve feasibility of virtual program delivery and maintain the reach and engagement of parents in the program amid the pandemic.

### Parent Program Implementation Support Adaptations

During the summer of 2020 and the spring of 2021, the IIK–IY Team safely offered in-person training for new Parent Program facilitators in accordance with state and local public health mandates. The Parent Program training content, materials, and activities were consistent with prior years, but there was time dedicated to discussing guidelines for how to deliver the program virtually, including how to set up and facilitate role plays, how to show the video vignettes, and how to use incentives with parents. Parent Program coaching was only available virtually. The setting shifted from in-person observations of the parent group followed by a coaching conversation between the IIK–IY Team member and the facilitators, to virtual observations and coaching of the parent group by the IIK–IY Team member who was joining from home.

Prior to the pandemic, the IIK–IY Team offered coaching to Parent Program facilitators based on their level of experience delivering the program. In anticipation of the steep learning curve for Parent Program facilitators delivering the Parent Program virtually, the team decided to offer coaching for all interested facilitators during the 2020–2021 program year. The IIK–IY Team also decided to augment their coaching support by providing more assistance to facilitators around preparation and planning, given all the logistics that needed to be considered to ensure a successful virtual parent group. A series of optional, virtual support groups with the IIK–IY Team were also available for Parent Program facilitators to join throughout the 2020–2021 program year so that they could troubleshoot challenges they experienced delivering the program in a virtual setting. The IIK–IY Team created resource videos so that facilitators could see examples of how to integrate core fidelity elements in the virtual setting, such as how to set up a virtual role play for participants. The IIK–IY Parent Program coaching protocol was tailored so that facilitators would receive coaching on all core fidelity elements when previously, the specific fidelity elements that the facilitators received coaching on depended on the facilitators’ levels of experience. A new point of emphasis was added to the coaching protocol, which involved supporting facilitators to intentionally connect with participants about their stress levels and self-care. Collectively, the goals of the IIK–IY implementation support adaptations for Parent Program were to improve feasibility of delivering the program virtually and ensuring the effectiveness of the program in the virtual context.

### Parent Program Evaluation Adaptations

Adaptations to the IIK–IY Parent Program process evaluation followed a similar procedure as to what was previously described for IIK–IY Dinosaur School. During the 2019–2020 program year and prior to the onset of the pandemic, the IIK–IY Team used the IIK–IY Parent Program fidelity checklist up to two times throughout each 14-week parent group to assess Parent Program facilitators’ fidelity to the program model. This unpublished checklist was previously developed by the IIK–IY Team. The Parent Program fidelity checklist consisted of 24 fidelity items that were rated on a 5-point Likert scale, with higher scores indicating a greater degree of fidelity. The score for each fidelity item was based on the number of guide statements the IIK–IY Team member observed as completed by the Parent Program facilitators during the coaching visit. Example items from the Parent Program fidelity checklist include “Parent Program facilitators use a variety of strategies that meet parent needs and keep parents engaged,” and “Parent Program facilitators ensure that homework discussion/wrap up occurs at the end of each session.” This checklist was also developed specifically for in-person program delivery. The IIK–IY Team made the decision to modify the Parent Program fidelity checklist in order to improve the fit of the checklist for virtually assessing Parent Program facilitators’ program fidelity for virtual and in-person program delivery.

The IIK–IY Team examined each of the Parent Program fidelity checklist items and the corresponding guide statements. For the few fidelity items and guide statements that did not fit both in-person and virtual delivery formats, the wording was either tailored so that the fidelity item or guide statement could still be evaluated regardless of program delivery format, or its applicability to only an in-person delivery format was recorded in the checklist. In addition, a handful of Parent Program fidelity items were reformatted as open-ended questions, in order to gather qualitative information about what Parent Program facilitators’ delivery of that fidelity item looked like for virtual and in-person program delivery.

The Parent Program fidelity checklist rating scale was revised in order to make it more feasible for the IIK–IY Team to rate Parent Program facilitators’ fidelity for in-person and virtual program delivery contexts. The updated rating scale was formatted to be consistent with the updated Dinosaur School fidelity rating scale, which included two dichotomous response options for each fidelity item, including whether the fidelity item was observed (“yes” or “no”), and the extent to which the fidelity item was delivered, if observed (“full” or “partial”). The updated Parent Program fidelity checklist consisted of 25 total items that were similar to the earlier version, with the revised rating scale being the most significant adaptation. See Online Resource 4 for a list of the 2020–2021 IIK–IY Parent Program fidelity items.

The IIK–IY process evaluation for Parent Program involves tracking the IIK–IY Team’s coaching visits with Parent Program facilitators. Two questions were added to the 2020–2021 visit tracking forms to capture information about the virtual coaching and program delivery format at each coaching visit. The first question was about the specific virtual coaching modality for the coaching visit (e.g., Zoom, Google Meets, Microsoft Teams, etc.) and the second question was about the program delivery format (in-person or virtual). The goal of adding these questions was to monitor Parent Program coaching visits by program delivery format.

In addition, Parent Program facilitators administer weekly surveys to participants in their group. These brief surveys give participants an opportunity to provide feedback about the extent to which program activities and content are helpful. For the 2020–2021 program year, the IIK–IY Team added a question about participants’ satisfaction with the virtual format. The purpose of adding this question was so that facilitators could use this feedback to improve the weekly program sessions and specifically identify whether there were any barriers to participating in the program virtually.

There were also a few adaptations to the IIK–IY Parent Program outcomes evaluation. Before the start of the 2020–2021 program year, the IIK–IY Data and Evaluation Manager determined that participants from both in-person and virtual groups would still be able to complete the same pre–post-outcome measures at the beginning and end of the 14-week parent group, and the program format would be tracked by group. The IIK–IY Parent Program pre–post-outcomes measures include 12 items that make up the Social Competence Scale-Parent (SCS-P) Report (Conduct Problems Prevention Research Group, 1995) and 68 items from the Parenting Practices Interview (PPI) survey (Webster-Stratton et al., 2001). Example items from the SCS-P include “My child does what he/she is told to do,” and “My child works out problems with friends or brothers and sisters on his/her own,” and examples from the PPI include “How often do you praise or compliment your child when your child behaves well or does a good job?,” and “How often do you give your child a time out when he/she misbehaves (that is, does something he/she is not supposed to do)?”. The SCS-P is rated by program participants on a 5-point Likert scale, with higher scores indicating a greater degree of social competence in their preschool-aged child. The PPI is completed by program participants on a 7-point Likert-type scale, indicating the frequency or likelihood with which they use a particular parenting strategy. The PPI outcome variables included in the IIK–IY outcomes evaluation include three positive summary scores (Positive Parenting, Appropriate Discipline, and Clear Expectations) and two negative summary scores (Harsh Discipline and Inconsistent Discipline). It is important to note that the PPI Harsh Discipline and Inconsistent Discipline summary scores are expected to show a decrease between the pre- and post-test, which reflects less frequent or lower likelihood of using those child rearing strategies. Conversely, Positive Parenting, Appropriate Discipline, and Clear Expectations are expected to show an increase between the pre- and post-test, which reflects more frequent or greater likelihood of using those types of skills.

The IIK–IY Team also added a couple of questions to the post-satisfaction survey about participants’ experiences with the virtual program. In the spring of 2021, a subgroup of the IIK–IY Team created a new survey for Parent Program facilitators that were designed to elicit feedback about their experiences delivering the program amid the ongoing pandemic. Facilitators were asked about the additional IIK–IY Parent Program implementation supports, what strategies they used to recruit and engage parents, and whether they found innovative ways to provide meals and childcare resources for families. Facilitators were also asked to provide qualitative feedback regarding their successes and challenges delivering the program in person and/or virtually during the 2020–2021 program year.

### Parent Program Outcomes and Fidelity

During the 2020–2021 program year, the IIK–IY Team supported 67 Parent Program facilitators who co-led 42 different parent groups, with 375 participants across 15 Colorado counties. See Online Resource 5 for demographic information about the Parent Program participants.

Based on results from the 2020–2021 IIK–IY Parent Program evaluation, Table 5 demonstrates the Parent Program outcomes by program delivery format. A paired samples *t*-test analysis revealed that, on average, there was a statistically significant difference between the pre- and the post-test for all Parent Program outcome variables, except for Appropriate Discipline for participants from in-person groups. On average, Parent Program participants who participated in a virtual or in-person parent group reported significantly higher levels of social competence in their children and higher levels of the Positive Parenting and Clear Expectations, and lower levels of Harsh Discipline and Inconsistent Discipline at the end of the group, compared to the beginning of the group. The only exception was that participants from in-person groups, on average, did not report a statistically significant difference in their use of Appropriate Discipline strategies at the end of the group compared to the beginning of the group. The size of the pre–post-difference for the PPI Clear Expectations, Harsh Discipline, and the Social Competence overall score was similar for parents who participated in virtual or in-person groups, on average, as all three scores were within a tenth of a point. There was a bit more variability for the Positive Parenting and Inconsistent Discipline scores according to program delivery format; on average, the Positive Parenting and Inconsistent Discipline difference scores were smaller for participants from in-person groups, and the size of the difference was less than a fifth of a point. The Appropriate Discipline difference score, on average, was approximately a third of a point smaller for participants from in-person groups. The adaptations to the Parent Program outcomes evaluation enabled IIK’s measurement of Parent Program participants’ outcomes accounting for the program context.

**Table 5:** 2020–2021 IIK–IY Parent Program participant outcomes by program delivery format

Outcome variable	Program delivery format^a^	*M* (SD)	*t*^b^	df	*p* value
Social Competence overall score (SCS-P)	Virtual	− 0.38 (0.67)	− 8.58	220	< .001
	In person	− 0.39 (0.83)	− 3.47	51	.001
PPI Appropriate Discipline	Virtual	− 0.47 (0.93)	− 7.73	233	< .001
	In person	− 0.16 (1.30)	− 0.90	53	.38
PPI Positive Parenting	Virtual	− 0.57 (0.82)	− 10.54	233	< .001
	In person	− 0.43 (0.79)	− 3.97	53	< .001
PPI Clear Expectations	Virtual	− 0.47 (1.23)	− 5.85	233	< .001
	In person	− 0.56 (1.29)	− 3.20	53	.002
PPI Harsh Discipline	Virtual	0.40 (0.71)	8.52	233	< .001
	In person	0.37 (0.69)	3.92	53	< .001
PPI Inconsistent Discipline	Virtual	0.51 (0.86)	9.15	233	< .001
	In person	0.33 (0.85)	2.85	53	.006

*SCS-P* Social Competence Scale-Parent Report; items rated on a scale of 1–5, *PPI* Parenting Practices Interview (survey); items rated on a scale of 1–7

^a^Program delivery format indicates the format by which the Parent Program facilitators delivered the program content

^b^Paired samples (pre–post) *t*-test results by program delivery format for the 2020–2021 IIK–IY Parent Program evaluation in Colorado

Content analysis of participants’ open-ended responses about their experiences with the program provides additional context for the quantitative outcomes. Participants from virtual and in-person groups emphasized their learning and growth in their confidence and abilities to apply several different parenting strategies (e.g., child-directed play, using praise and incentives) as being particularly helpful. They also appreciated the discussions and hearing the perspectives of other parents that were experiencing similar challenges, and they appreciated the insights and guidance of the facilitators in a nonjudgmental, compassionate environment. Participants also indicated that the topics covered, and related program resources and materials were relevant and useful. In terms of how the program could be changed to better help participants, some suggested that it would have been helpful to spend additional time discussing certain topics. Many virtual group participants also mentioned that while they enjoyed the program, it would have been better to meet with the group in person and benefit from face-to-face interactions and discussions. The overwhelming majority reported that they would participate in virtual sessions again, although several people noted that they would prefer in-person sessions.

The documentation of Parent Program facilitators’ successes and challenges delivering the program during the 2020–2021 program year offered another perspective on the program adaptations. A content analysis of facilitators’ open-ended responses revealed that they perceived a high level of participant engagement in the virtual sessions, as well as high rates of attendance and retention. They also noted that participants were able to create supportive communities within groups, even virtually. Some facilitators emphasized participants’ growth in their use of the different parenting practices and noted that participants provided positive feedback about the group. A few mentioned that the virtual format increased the accessibility of the program for families who would have otherwise been unable to participate in person.

Though several facilitators considered participant engagement to be high, it was also identified as a challenge for virtual groups. Facilitators from a few groups noted that getting each participant to leave their camera on was difficult, and participants tended to get distracted during the sessions by what was going on at home. Relatedly, another barrier to supporting the program virtually was the lack of childcare for facilitators and participants. Several facilitators referenced the challenge of setting up role plays, a fidelity element, in virtual settings. Feedback from facilitators who supported participants from rural Colorado counties highlighted the urban versus rural digital divide, as these virtual groups struggled with unreliable internet connections. A few facilitators also commented on variability in participants’ comfort levels with technology and in using online meeting platforms. Lastly, some explained that it felt harder to build relationships with participants in virtual groups, compared to in-person settings. The Parent Program facilitator feedback suggests that overall, the key challenge was to deliver the virtual program with fidelity; however, facilitators also seemed to see a lot of success in terms of participants’ growth in their parenting skills. Participants’ feedback indicated that they enjoyed and greatly benefitted from the groups, regardless of program delivery format. This is largely consistent with the quantitative participant pre–post-outcomes.

The adaptations to the 2020–2021 Parent Program fidelity checklist allowed the IIK–IY Team to virtually assess Parent Program facilitators’ fidelity up to two times during the 14-week group for virtual and in-person program delivery. Overall, there were far fewer facilitators that were observed doing in-person Parent Program during the 2020–2021 program year compared to the number of facilitators that were observed delivering the program virtually. On average, the IIK–IY Team observed 79% of fidelity items being delivered by facilitators of in-person groups at checklist one and 86% of fidelity items at checklist two. On average, the IIK–IY Team observed 86% of fidelity items being delivered by facilitators of virtual groups at checklist one, and 88% of fidelity items, on average, at checklist two. See Table 6 for the mean number of Parent Program fidelity items observed (i.e., *“*yes*”* for that fidelity item) by program delivery format. It is interesting that the mean number of fidelity items that the IIK–IY Team observed was lower for facilitators of in-person groups compared to virtual groups. This pattern generally aligns with the Parent Program outcomes; some of the outcomes for in-person participants were slightly smaller than the outcomes for virtual program participants.

**Table 6:** 2020–2021 Mean number of IIK–IY Dinosaur School fidelity items observed by program delivery format

Program delivery format^a^	Checklist 1^b^		Checklist 2	
	*n*^c^	*M*	*n*	*M*
Virtual	18	18.06	15	18.47
In person	2	16.50	2	18.00

^a^Program delivery format indicates the format the facilitators were using to deliver the program when each fidelity checklist was completed

^b^There were a total of 21 items on the IIK–IY Parent Program Fidelity Checklist included in the mean fidelity ratings

^c^Refers to the number of Parent Program facilitator pairs for whom the IIK–IY Team had completed fidelity checklists

### Summary and Lessons Learned

During the 2020–2021 program year, the IIK–IY Team partnered with Colorado communities in 20 counties to support the delivery of Dinosaur School and Parent Program for 4027 students, 315 teachers, 67 Parent Program Facilitators, and 375 Parent Program participants. The IIK–IY Team successfully adapted their implementation supports, coaching teachers and Parent Program facilitators virtually, and supported many teachers and facilitators with virtual program delivery so that children and families in Colorado could continue to benefit from IY programming. Prior to the onset of the pandemic, the IIK–IY Team had not considered supporting teachers or Parent Program facilitators with virtual program delivery, as both programs were developed for in-person delivery. The shift to a virtual world necessitated a contextual change to Dinosaur School and Parent Program delivery, and a change to the mode of the IIK–IY Team’s training and coaching. Without the use of the FRAME tool and the contextual adaptations made to the IY programs and IIK’s implementation supports, it would have been much harder, if not impossible, for IIK’s partner agencies to effectively deliver Dinosaur School and Parent Program throughout the pandemic. The IIK–IY Team’s adaptations helped to ensure that the IY programs continued to be usable innovations (Blase et al., 2018).

There are four main criteria for usable innovations, including a clear description of the innovation, its essential functions, operational definitions of the essential functions, and a pragmatic way to assess fidelity (Blase et al., 2018). In this case, the theoretical underpinnings for the IY programs and their essential functions were still intact. The FRAME tool provided a comprehensive, organized structure so that the IIK–IY Team could operationalize the essential functions of IY in the context of their adaptations. The tool also informed the refinement of the IIK–IY Team’s fidelity assessments across the adapted program contexts. The evidence gathered as part of the 2020–2021 IIK–IY evaluation and within a decision support data system suggested that teachers and Parent Program facilitators were able to maintain high-quality use of Dinosaur School and Parent Program in Colorado during the COVID-19 pandemic. It also revealed that virtual program delivery may be a suitable option for some Colorado communities, even when the pandemic is over.

A central focus that guided the IIK–IY Team’s response to the pandemic was to make adaptations while also promoting a high level of fidelity to the program. There were adaptations to the IIK–IY fidelity checklists, intended to make it more feasible for the IIK–IY Team to assess teacher and facilitators’ program delivery across contexts. An unintended consequence was that one of the checklist adaptations made it more challenging to understand what high Dinosaur School and Parent Program fidelity looked like. In particular, collapsing the rating options from a 5-point Likert scale to two categories (full versus partial delivery) made it harder to gauge the extent to which each item was delivered during the fidelity observation. However, IIK–IY coaching and fidelity observations continued to be informative for the IIK–IY Team in identifying what supports were needed and defining expectations about reasonable next steps to support teachers’ and Parent Program facilitators’ fidelity in the midst of a pandemic. Despite less clarity and precision about the degree to which fidelity was adhered to throughout the program year, IIK–IY’s decision support data system and other Competency and Organizational Implementation Drivers helped to compensate.

A limitation of this case study is that the IIK–IY Dinosaur School and Parent Program fidelity and outcomes information presented does not assess the extent to which the various adaptations individually or collectively affected program fidelity and outcomes, and thus any inferences about the links between the program adaptations and the data points presented here are extremely limited. However, this case study contributes important information to the implementation science and practice literature about what large-scale, statewide adaptations to an EBP look like in community-based settings and describes the proximal program outcomes and fidelity in the context of such adaptations. Additionally, this case study provides an in-depth look at the application of an implementation science tool (FRAME; Stirman et al., 2019) that served as an anchor for a process of making pandemic-related adaptations by the IIK–IY Team at a Colorado, USA-based intermediary organization. The nine guiding questions from the FRAME served as a strong foundation for an intentional and purposeful process that involved thoughtful planning, comprehensive tracking of various adaptations, and careful monitoring of program outcomes and fidelity. As part of an intermediary organization with a robust decision support data system in place, the IIK–IY Team was able to integrate and use the FRAME seamlessly within the existing scope of implementation support work.

## Supplementary Information

Below is the link to the electronic supplementary material.

Supplementary file1 (PDF 22 kb)

Supplementary file2 (PDF 14 kb)

Supplementary file3 (PDF 21 kb)

Supplementary file4 (PDF 15 kb)

Supplementary file5 (PDF 20 kb)
