# Supplementary material for: Adaptations in the Context of COVID-19: Application of an Implementation Science FRAMEwork
Source: Glob Implement Res Appl. 2022 Jun 27;2(4):278–92. doi: 10.1007/s43477-022-00048-1 (PMC9243998; doi:10.1007/s43477-022-00048-1)
Supplement: Supplementary file 1 — Supplementary file1 (PDF 22 kb) [file 43477_2022_48_MOESM1_ESM.pdf]

Article Title: Adaptations in the Context of COVID-19: Application of an Implementation Science FRAMEwork
Journal Name: *Global Implementation Research and Applications*
Author Names: Erin C. Albrecht, Lindsay Sherman, Amanda Fixsen, and Julie Steffen
Affiliation and e-mail address of corresponding author: Invest in Kids, ealbrecht@iik.org

Online Resource 1

Examples of the IIK-IY Implementation Drivers

Implementation drivers	Examples of IIK-IY supports
Competency drivers<br>(Selection, Training, Coaching, and Fidelity)	• Guidance selecting staff for specialized roles (e.g., Parent Program facilitators, support staff for ancillary needs related to training and Parent Program)<br>• Ongoing training, coaching, and technical assistance for both long-time and newly-trained implementers<br>• Cultivation of internal coaching expertise at the site level through leadership and coaching and training alongside the IIK-IY Team<br>• Ongoing fidelity assessment by the IIK-IY Team to inform coaching, training, and outcomes
Organizational drivers<br>(Decision Support Data System, Facilitative Administration, and Systems Intervention)	• Annual pre and post data collection for students, teachers, and parents served by the program, along with outcome reports for partner agencies, districts, and counties<br>• Development of a Local Implementation Team (LIT) at each partner agency consisting of direct implementers, site leadership, community partners, and an IIK-IY team member<br>• Ongoing assessment of site and community level implementation strengths, opportunities, and related goal setting<br>• Financial as well as structural support to engage community partners in IY expansion and referral partnerships
Leadership Drivers (Technical and Adaptive)	• Proactive communication with site leadership to support them in anticipating, identifying, and working through challenges<br>• Ongoing support to site leadership to strengthen implementation drivers
