# Supplementary material for: Adaptations in the Context of COVID-19: Application of an Implementation Science FRAMEwork
Source: Glob Implement Res Appl. 2022 Jun 27;2(4):278–92. doi: 10.1007/s43477-022-00048-1 (PMC9243998; doi:10.1007/s43477-022-00048-1)
Supplement: Supplementary file 2 — Supplementary file2 (PDF 14 kb) [file 43477_2022_48_MOESM2_ESM.pdf]

Article Title: Adaptations in the Context of COVID-19: Application of an Implementation Science FRAMEwork
Journal Name: *Global Implementation Research and Applications*
Author Names: Erin C. Albrecht, Lindsay Sherman, Amanda Fixsen, and Julie Steffen
Affiliation and e-mail address of corresponding author: Invest in Kids, ealbrecht@iik.org

Online Resource 2

2020-2021 IIK-IY Dinosaur School Fidelity Items

1. Teacher uses developmentally appropriate content in large group
2. Teacher clearly communicates the goals/objectives of large group
3. Teacher facilitates or teaches the large group lesson in the right sequence for that time of year
4. Teacher facilitates small group activity
5. Teacher uses developmentally appropriate content in small group activity
6. Teacher has a communication bridge between home and school in place
7. Teacher uses IY vignette(s) during Dinosaur School
8. Teacher positively integrates the Dinosaur School puppets into the classroom culture
9. Teacher integrates Dinosaur School visuals and/or social-emotional visuals and materials into their large group Dinosaur School lessons
10. Teacher integrates Dinosaur School visuals and/or social-emotional visuals and materials into activities outside of Dinosaur School (e.g. recess, center time)
11. Teacher uses positive reinforcement strategies during observation (discipline hierarchy)
12. Teacher encourages child engagement during observation
13. Co-teacher supports Dinosaur School during the observation (leave item blank if there is no co-teacher)
14. Teacher promotes use of Dinosaur School language and skills during the large and small group lessons
15. Teacher creates opportunities for children to practice Dinosaur School language and skills in the lesson (large and/or small group)
16. Teacher uses role play during Dinosaur School
17. The teacher coaches children to use Dinosaur School skills during challenging behavior
18. Teacher integrates real classroom or home issues into Dinosaur School lessons (large and/or small group)
19. How is the teacher ensuring that Dinosaur School visuals and/or other social-emotional visuals and materials are visible?
20. How is the teacher promoting the use of Dinosaur School language and skills outside of the lesson?
