# Supplementary material for: Adaptations in the Context of COVID-19: Application of an Implementation Science FRAMEwork
Source: Glob Implement Res Appl. 2022 Jun 27;2(4):278–92. doi: 10.1007/s43477-022-00048-1 (PMC9243998; doi:10.1007/s43477-022-00048-1)
Supplement: Supplementary file 3 — Supplementary file3 (PDF 21 kb) [file 43477_2022_48_MOESM3_ESM.pdf]

Article Title: Adaptations in the Context of COVID-19: Application of an Implementation Science FRAMEwork
Journal Name: *Global Implementation Research and Applications*
Author Names: Erin C. Albrecht, Lindsay Sherman, Amanda Fixsen, and Julie Steffen
Affiliation and e-mail address of corresponding author: Invest in Kids, ealbrecht@iik.org

Online Resource 3

2020-2021 Demographic Characteristics of Dinosaur School Students and Teachers

	Students (*n* = 4,027)		Teachers (*n* = 315)	
Characteristic	n	%	n	%
Gender				
Male	2,043	50.7	5	1.6
Female	1,984	49.3	308	97.8
Declined to respond	-	-	2	0.6
Race/ethnicity				
Non-Hispanic White	1,763	43.8	206	65.4
Hispanic/Latino	1,668	41.4	80	25.4
Multiracial	296	7.4	10	3.2
Black/African-American	180	4.5	5	1.6
Asian	83	2.1	2	0.6
American Indian/Alaska Native	21	0.5	1	0.3
Native Hawaiian and Other Pacific Islander	16	0.4	1	0.3
Other race/ethnicity	-	-	1	0.3
Declined to respond	-	-	9	2.9
Grade				
Pre-K/Preschool	3,212	79.8	276	87.6
Kindergarten	735	18.3	33	10.5
First Grade	80	2.0	6	1.9
Age (years)	*Mdn*[a] = 4 (2-7)		*Mdn* = 43 (19-73)	

[a]*Mdn* indicates the median age in years. The numbers in parentheses indicate the range of ages from the lowest to the highest.
