# Supplementary material for: Adaptations in the Context of COVID-19: Application of an Implementation Science FRAMEwork
Source: Glob Implement Res Appl. 2022 Jun 27;2(4):278–92. doi: 10.1007/s43477-022-00048-1 (PMC9243998; doi:10.1007/s43477-022-00048-1)
Supplement: Supplementary file 4 — Supplementary file4 (PDF 15 kb) [file 43477_2022_48_MOESM4_ESM.pdf]

Article Title: Adaptations in the Context of COVID-19: Application of an Implementation Science FRAMEwork
Journal Name: *Global Implementation Research and Applications*
Author Names: Erin C. Albrecht, Lindsay Sherman, Amanda Fixsen, and Julie Steffen
Affiliation and e-mail address of corresponding author: Invest in Kids, ealbrecht@iik.org

Online Resource 4

2020-2021 IIK-IY Parent Program Fidelity Items

1. Parent Program Facilitators (PPFs) prepare a welcoming environment
2. PPFs post and refer to visuals during the group
3. PPFs present new learning
4. PPFs facilitate homework discussion at the beginning of the session that supports learning and skill-building
5. PPFs use praise
6. PPFs deliver curriculum accurately across sessions
7. PPFs use their child development knowledge to support parents
8. PPFs ensure that homework discussion/wrap up occurs at the end of each session
9. PPFs use a variety of strategies that meet parent needs and keep parents engaged
10. PPFs reinforce new learning using a collaborative process
11. PPFs work effectively as a team
12. PPFs chart and reference key principles
13. PPFs have positive regard for parents and children
14. PPFs manage parent questions and concerns
15. PPFs use vignettes to demonstrate new skills
16. PPFs tailor the vignettes to meet the needs of the parents in the group
17. PPFs use vignettes to facilitate discussion
18. PPFs use planned role play
19. PPFs manage planned role play
20. PPFs use debrief after planned role plays
21. PPFs use spontaneous role plays
22. How do PPFs use incentives?
23. What does childcare delivery look like?

24. How do PPFs create space for participant engagement outside of the scheduled sessions (e.g., private meetings, use chat function for participants to engage before the session, follow-up emails and phone calls, etc.)?
25. What are the PPFs doing with the mealtime funds (e.g., providing gift certificates, sending meals each week to participants, etc.)?
