# Supplementary material for: Adaptations in the Context of COVID-19: Application of an Implementation Science FRAMEwork
Source: Glob Implement Res Appl. 2022 Jun 27;2(4):278–92. doi: 10.1007/s43477-022-00048-1 (PMC9243998; doi:10.1007/s43477-022-00048-1)
Supplement: Supplementary file 5 — Supplementary file5 (PDF 20 kb) [file 43477_2022_48_MOESM5_ESM.pdf]

Article Title: Adaptations in the Context of COVID-19: Application of an Implementation Science FRAMEwork
Journal Name: *Global Implementation Research and Applications*
Author Names: Erin C. Albrecht, Lindsay Sherman, Amanda Fixsen, and Julie Steffen
Affiliation and e-mail address of corresponding author: Invest in Kids, ealbrecht@iik.org

Online Resource 5

2020-2021 Demographic Characteristics of Parent Program Participants and Facilitators

	Participants (n = 375)		Facilitators (n = 67)	
Characteristic	n	%	n	%
Gender				
Male	64	16.4	5	7.5
Female	299	76.7	57	85.1
Other	1	0.3	-	-
Missing	-	-	5	7.5
Race/ethnicity				
Non-Hispanic White	153	39.2	33	49.3
Hispanic/Latino	180	46.2	24	32.8
Multiracial	18	4.6	-	-
Black/African-American	6	1.5	-	-
Asian	9	2.3	-	-
American Indian/Alaska Native	1	0.3	-	-
Native Hawaiian and Other Pacific Islander	1	0.3	1	1.5
Missing	-	-	9	13.4
Group language				
Spanish	136	34.9	22	32.8
English	254	65.1	44	65.7
